# Spatial Difference of Transit-Based Accessibility to Hospitals by Regions Using Spatially Adjusted ANOVA

**DOI:** 10.3390/ijerph16111923

**Published:** 2019-05-30

**Authors:** Meijie Chen, Yumin Chen, Xiaoguang Wang, Huangyuan Tan, Fenglan Luo

**Affiliations:** 1 School of Resource and Environment Science, Wuhan University, Wuhan 430079, China; Chen_meijie@whu.edu.cn (M.C.); tanhuangyuan@whu.edu.cn (H.T.); FengLan_Luo@whu.edu.cn (F.L.); 2 Department of Geography, Central Michigan University, 287 Dow Science Complex, Mount Pleasant, MI 48859, USA; Wang9x@cmich.edu

**Keywords:** ANOVA, Spatial difference, spatial lag model, accessibility

## Abstract

This paper proposes a spatial difference analysis method for evaluating transit-based accessibility to hospitals using spatially adjusted ANOVA. This method specializes in examining spatial variations of accessibility to hospitals by regions (i.e. administrative districts or subdistricts). The spatial lag model is applied to adjust traditional ANOVA, which reduces spatial dependency and avoids false rejection to null hypothesis. Multiple comparison methods are used for further detection of differences in accessibility between regions. After multiple comparison, accessibility within regions is classified into three levels. The study is conducted on two scales—administrative districts and subdistricts—to discuss spatial variations in macro and micro dimensions respectively in the central part of Wuhan, China. Accessibility is calculated by using a simple model and a gravity model. The final classification results showed that the spatially adjusted method is more reliable than the traditional non spatially adjusted one and the gravity model can better detect more hidden information about the inequal distribution of medical resources. It is also found that the subdistricts, which have significantly lower accessibility to hospitals than others, are mainly distributed in Hongshan and Qingshan district. Our study hopes to shed new lights in spatial difference analysis for accessibility and provide policy recommendations that would promote equality in provisions of public health services.

## 1. Introduction

Hospitals are playing an important role in maintaining people’s health. It is particularly important for health professions, researchers, and planners to know how well the public health facilities serve the public. A rational distribution of hospitals could ensure people to have equal access to health care and help the government to achieve the goal of equalization of public health services. Public transit, with low cost and high energy efficiency, has become one of the most important travel modes in people’s daily life, especially in countries where cities are rapidly urbanizing and the mobility challenge is daunting. To achieve sustainability, governments in different parts of the world are concentrating investments in public transport infrastructure [1]. It is more important now than ever to evaluate transit-based accessibility to hospitals. This study aims to develop a method to analyze the spatial variations of transit-based accessibility to hospitals, which would provide insights in promoting a distribution of health facilities that are equal and efficient. 

Accessibility is defined as the ease for people to overcome spatial separation or the potential of opportunities for interaction [2]. There are many definitions for accessibility to healthcare, two of which are mentioned most frequently in geographical analysis: (1) the number of opportunities available to a population; (2) the impedance between patient location and supply points [3]. Various methods were proposed for measuring accessibility, all of which have different focuses. For instance, Dutt et al. measured accessibility by taking the average of travel impedance (distance or time) to hospitals [4]. This method has two obvious shortcomings, which includes overestimating the influence of peripheral hospitals within the study area as well as ignoring the relationship between the demand of health series and supply ability. Another popular method is gravity models firstly proposed by Hansen [2]. Compared to other measures, the gravity models are superior as they consider the number of opportunities and travel impedance at the same time [5,6]. Variations of gravity model were developed later which includes two-step floating catchment area method (2SFCA). This model defines a service area by a threshold of travel time as well as taking supply side and demand side into consideration. A lot of researchers had used 2SFCA and its modified models to measure healthcare access [7,8,9,10]. However, as 2FSCA model needs to determine the proper catchment threshold which tends to be arbitrary and difficult. 

For transportation equity has become an important factor in the process of city development, the analysis for spatial disparity of accessibility has become a hot issue. Some studies employ traditional descriptive statistics and mapping to analyze the spatial patterns among areas [11,12,13]. Although these studies can intuitively describe the distribution characteristics and variation levels within data, they tend to be descriptive and exploratory. Some scholars used z-scores and its corresponding Low-High map to determine spatial disparity in accessibility [9,14,15]. This method could roughly identify whether accessibility within a subdistrict is above the standard or not as well as indicating accessibility-population mismatch, but it cannot determine which subdistrict has significantly higher or lower accessibility. Other researchers add spatial autocorrelation into consideration to further discuss the spatial pattern of accessibility. One of the commonly used methods for the detection of spatial patterns is LISA (local indicators of association), which can identify spatial clusters with high and low values, and thus uncover spatial outliers and uneven resource distribution [16,17,18,19]. Though studies above provided a substantial contribution to accessibility analysis, several issues need to be further considered. For example, LISA could not be used to uncover the spatial disparity between units that are not clustering. More researches are much needed to detect spatial variations and compare spatial differences across regions. 

Analysis of variance (ANOVA) is a traditional but effective way to measure whether regional means come from the same population, or more specifically, whether the means among groups are significantly different. A few scholars applied the ANOVA techniques in spatial difference analysis [20,21]. However, most geographical data are spatially autocorrelated and studies above did not take it into consideration. If a spatial dependency exists, observations could carry duplicated information, making effective sample size much smaller than it looks like and resulting in wrong rejection to null hypothesis [22,23]. Some scholars provide possible adjustments to the analysis of variance model such as calculating effective sample size or applying spatially autocorrelated error terms for liner regression [24,25,26,27]. Unfortunately, the implication of these modified models in spatial difference analysis is still very few, especially in the field of transportation.

Enlightened by the studies above, we aim to apply a spatially adjusted ANOVA to analyze the spatial difference of transit-based accessibility to hospitals. The central part of Wuhan, China is taken as an experimental area. Our goal is to examine if public transit service resources and medical care resources are equally distributed among regions, and discover the spatial differences. Spatial lag model is applied to eliminate the influence of spatial autocorrelation on hypothesis test. Multiple comparison methods will be used for further detection of the variation between groups. With the comparison results, accessibility could be classified into different levels. In addition, the study compares accessibility on two geographic scales: the administrative district (the macro scale) and the subdistrict (the micro scale) to uncover more details about medical resources distribution. We also calculate two types of accessibility measures using the simple model and the gravity model. By procedures above, three major questions could be answered: (1) What is the merit of spatially adjusted ANOVA and why it could perform better than the nonspatial one? (2) Do residents in central Wuhan have equal access to 3A hospitals under public transit travel mode and which spatial units have significantly higher or lower accessibility to hospitals? (3) What is the difference between the accessibility result calculated by simple model and gravity model?

The rest of this paper is organized as follows. Section 2 introduces study areas, data resources and several experimental methods. Section 3 presents the results of accessibility calculation and spatial difference analysis on two study scales. Section 4 includes discussions about the existing inequity discovered through results above and the corresponding suggestions. Section 5 draws conclusions and discusses the ideas for future study.

## 2. Materials and Methods 

### 2.1. Study Area

**Figure 1:**
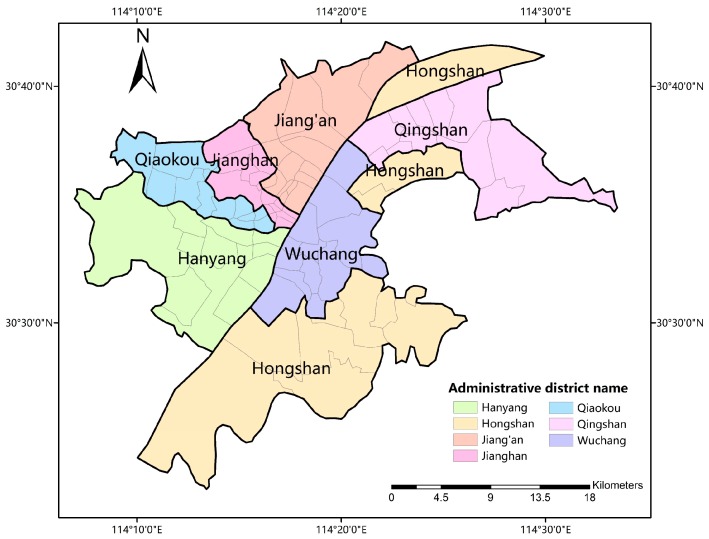
The study area.

Wuhan, with its extension of longitude 113°41’ to 115°05’, latitude 29°58’ to 31°22’, is the largest city in central China. Until 2011, there were 7061 buses and 289 bus routes with average length of 19.9 kilometers. This makes public transport one of the most important travel mode in citizen’s daily life. Considering data availability and study priority, we take central Wuhan as a case study (Figure 1). This region includes 86 sub-districts under the jurisdiction of 7 administrative districts (Jiang’an, Jianghan, Qiaokou, Hanyang, Wuchang, Qingshan, Hongshan) and is the main area for residents’ medical, educational and economical activities. 

### 2.2. Data Resources

#### 2.2.1. Transit Data

Travel distance and travel time are two main factors in the assessment of accessibility. In transit-based accessibility to hospitals analysis, bus stops and tertiary level-A hospitals (denoting as 3A hospital in the following paper) are taken as start points and target points respectively. Instead of using road network data and speed limit to calculate theoretical travel distance and time, Baidu Map API (http://lbsyun.baidu.com/) is applied to obtain real time travel distances and travel times under transit mode between areas. The locations of bus stops and hospitals can also be harvested by Baidu Map API.

**Figure 2:**
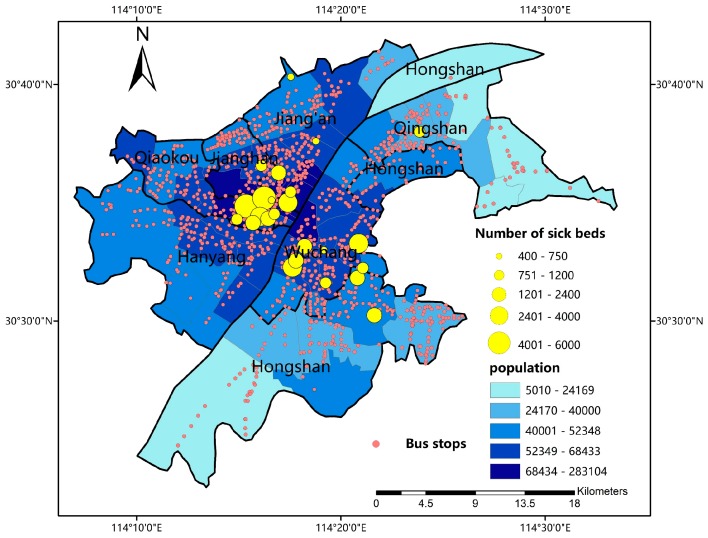
The distribution of bus stops, 3A hospitals and population.

As traffic jam is not a certain event that happens every day and detecting spatiotemporal fluctuation of traffic condition is not our main purpose in this paper, the code was run after 9pm to avoid outliers caused by traffic jam, therefore, to indicate the equity of basic medical resource distribution. As there are 1256 bus stops and 24 3A hospitals in study area, 30144 transit routes in total are included. The locations of bus stops and 3A hospitals are shown in Figure 2.

#### 2.2.2. Hospital Data and Population Data

In China, tertiary hospitals, especially 3A hospitals, contain the most important medical resources [15]. Therefore, in this study, we focus mainly on 3A hospitals. The number of sick beds in each hospital, which could be found at their official websites, are taken as an indicator of supply ability. Population data of each sub-district is obtained in census data 2014 and could be taken as an indicator of study units’ demand scale. The population data and the number of sick beds is also shown in Figure 2.

### 2.3. Methods

#### 2.3.1. Group Design

As our aim is to apply ANOVA to analyze the spatial variations between different groups, we need to determine study groups and minimum spatial units within each group. Study groups are defined on different scales: the administrative district (the macro scale) and the subdistrict (the micro scale).

**Figure 3:**
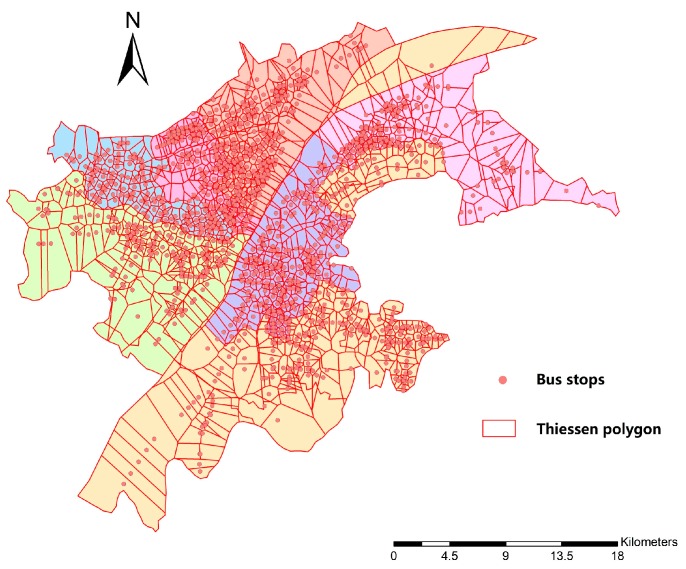
Thiessen polygons created by bus stops (1256 polygons).

**Figure 4:**
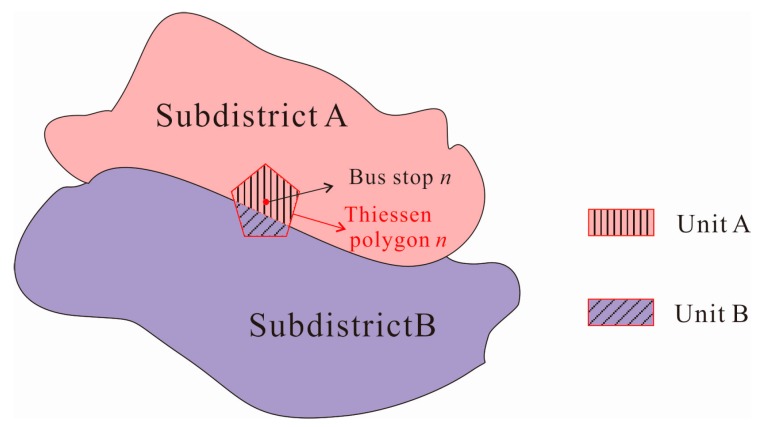
The division of minimum spatial units on subdistrict scale.

**Figure 5:**
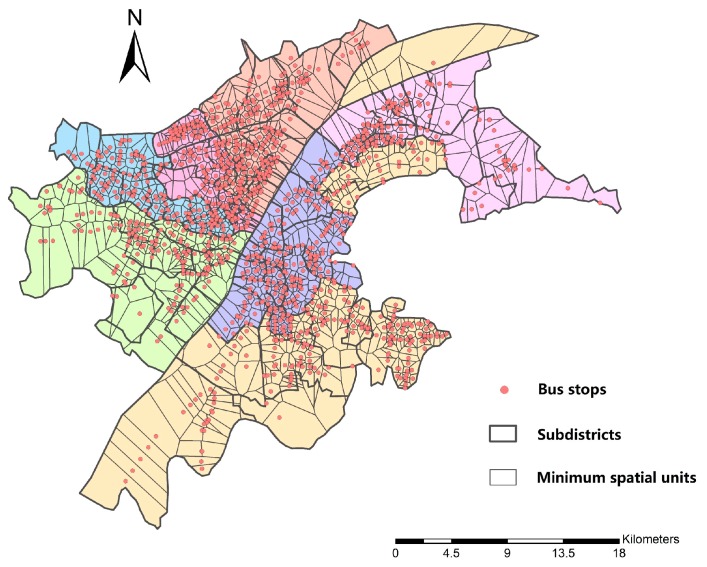
The minimum spatial units on subdistrict scale (2170 minimum spatial units).

On macro scale, seven administrative districts are viewed as study groups and 86 subdistricts within are taken as minimum spatial units within groups. While on micro scale, 86 subdistricts are taken as study groups and the theory of Thiessen polygon is adopted for the division of minimum spatial units, for any point in a Thiessen polygon has the closest distance to the corresponding polygon center [28]. 1256 bus stops are taken as center points to create 1256 corresponding Thiessen polygons (Figure 3). We assume that people whose location falls within that polygon tend to choose the only bus stop within and the polygon’s attribute (travel distance and travel time to hospitals) could be represented by the corresponding bus stop. Polygons that fall in each subdistrict are viewed as minimum spatial units of that group. For the polygons that fall on the boundary of two subdistricts, they are divided into two units (Figure 4). Unit A belongs to subdistrict A and its population density is the same as it is in subdistrict A. Same as unit B. The travel time to hospitals in unit A and B is the same, representing by bus stop n. After the division, there are 2170 minimum spatial units within 86 study groups (Figure 5).

#### 2.3.2. Accessibility Measurement

The measurements for transit-based accessibility to hospitals is operated in two different ways. One is a simple model proposed by [29], which can be demonstrated as follows:

(1) $$A_{i}=\frac{1}{n}\overset{n}{\underset{j=1}{\sum }}d_{ij},\;i=1,2,\ldots ,k$$

(2) $$E_{m}=\frac{1}{N}\overset{N}{\underset{i=1}{\sum }}A_{i},\;i=1,2,\ldots ,N$$

$A_{i}$ represents the accessibility to hospitals of i th bus stop. *n* (*n* = 24) is the number of 3A hospitals within the whole area. *k* (*k* = 1256) is the total number of bus stops within study area. $d_{ij}$ is defined as the travel cost between bus stop i and hospital j, usually being travel time or distance. As travel time is a reliable indicator of accessibility which could reflect the actual traffic condition, it is chosen to represent travel impedance in our study [30,31]. $E_{m}$ is denoted as each sub-region’s overall accessibility while N is the number of bus stops within corresponding sub-region *m*. The larger $E_{m}$ is, the less convenient the region could be. The simple model can reflect the basic transit service resources to 3A hospitals within the whole study area.

Another model is a modified gravity model proposed by Joseph and Bantock [5]. It adds travel impedance as a weight indicator, taking both demand side and supply side into consideration. The model can be shown in the following formulas:

(3) $$A_{i}=\overset{n}{\underset{j=1}{\sum }}\frac{S_{j}*d_{ij}^{-\beta }}{V_{j}}$$

(4) $$V_{j}=\overset{m}{\underset{k=1}{\sum }}D_{k}*d_{kj}^{-\beta }$$

Same as in Equations (1) and (2), $A_{i}$ and $d_{ij}$ represents accessibility and travel cost respectively. β is a gravity decay coefficient. According to Kwan [32], when β = 2 the gravity model could generate a more pronounced accessibility patterns, therefore we set β = 2 in our study. $S_{j}$ is supply scale of hospital j which is represented by the number of sick beds in this paper. $V_{j}$ represents the demand on hospital j and is calculated by summing up impedance weighted population of all spatial units, which could be seen in formula (4). $D_{k}$ is the demand scale of spatial unit k and in this paper, is represented by population. The integer n is the total number of hospitals within study area and m is the total number of spatial units.

#### 2.3.3. Spatially Adjusted ANOVA

ANOVA is a method to compare the means of variables among groups and to determine whether they are significantly different. If the variation between groups is much greater than variations within groups, the difference between groups is significant. Otherwise, the difference between means might just be due to sampling error. 

In ANOVA, there are three assumptions for sampling data: normality, homoscedasticity, independence. According to previous study, traditional one-way ANOVA can be rewritten as linear regression model using following equation [25]:

(5) $$Y^{*}=X\beta +\varepsilon$$

where $Y^{*}$ is the n×1 vector representing some characteristics, which should be independent; X is a n∗m binary dummy variable matrix transformed by factor variables, denoting which group the corresponding areal units belong to; ε is a n×1 vector representing random error term, which should be normally distributed and uncorrelated.

However, according to the first law of geography, variables are often spatially autocorrelated [33]. This paper uses spatial weights matrix and Moran’s I index to detect spatial autocorrelation. There are many ways to construct a spatial weights matrix W. In our study, the queen-contiguity case is chosen, which denote spatial units that share vertices or boundaries as contiguity.

With data spatially autocorrelated, the regression model could be developed by considering the presence of spatial autocorrelation in its spatial lag terms:

(6) $$Y=\rho WY+Y^{*}$$

*ρ* is an unknown parameter which reflects the spatial autocorrelation; WY is the spatial lag factor representing the influence of neighboring units. $Y^{*}$ is the independent part in accessibility Y.

Equation (6) yields

(7) $$Y^{*}=Y-\rho WY$$

Anselin proposed a maximum likelihood method for estimating *ρ* in spatial lag model [34]. Therefore, the influence of spatial autocorrelation in Y could be filtered out and the spatially adjusted ANOVA could be rewritten in regression form as:

(8) (I−ρW)Y=Xβ+ε

#### 2.3.4. Multiple Comparison for The Detection of Spatial Difference

Before conducting ANOVA, Shapiro-Wilk test and Levene’s Test are performed to examine if accessibility data meets the assumption of normality and homoscedasticity. Box-cox transformation, root transformation or logarithm should be taken if the data is not normally distributed [35]. If the data cannot be transformed into normally distributed one, another nonparametric test called Kruskal-Wallis test (also called one-way ANOVA on ranks) could be taken as an alternative. 

After appropriate data transformation, following the steps in Section 2.3.3 to filter out the influence of spatial autocorrelation. If the differences of ANOVA are significant at a certain level, at least one group has significant higher or lower accessibility to hospitals compared with other groups. 

To further identify which groups are indeed have significant higher or lower accessibility, multiple comparison methods are applied. One is Scheffe’s method [36], which is often used for the post hoc testing after ANOVA when the number of observations among groups are not equal. Another method is Dunn’s test [37], which is a commonly used non-parametric pairwise comparison after Kruskal-Wallis test. 

The results of Scheffe’s method and Dunn’s test both records *p*-value for each comparison pairs. However, it is difficult to identify if accessibility within a study group is significantly good or bad simply by *p*-values among comparison pairs. Therefore, we should compare not only *p*-value between subdistricts but also the average accessibility (denoting as $a_{i}$) within. Take spatial unit i in gravity model as an example, if $D_{ij}$ < 0.05 and $a_{i}$ > $a_{j}$, it means unit i has significantly higher average accessibility score than unit j and have better accessibility. Vice versa. Note that in simple model, the smaller the calculated accessibility $a_{i}$, the less the average the average travel time and the better the accessibility. Count the number of other subdistricts that subdistrict i is significantly higher or lower to and denote the number as “high” or “low”. A simple but useful algorithm is proposed to better illustrate the whole procedure (see Algorithm 1).


**Algorithm 1**
Simple model (the less the average travel time, the better the accessibility) for i = 0, 1, 2, …, 85  high=0, low=0  for j = 0, 1, 2, …, 85   if $D_{ij}$ < 0.05 & $a_{0}$ < $a_{j}$    high=high+1    else ($D_{ij}$ < 0.05)    low=low+1 Gravity model (the higher the accessibility score, the better the accessibility) for i = 0, 1, 2, …, 85  high=0, low=0  for j = 0, 1, 2, …, 85   if $D_{ij}$ < 0.05 & $a_{0}$ > $a_{j}$     high=high+1   else ($D_{ij}$ < 0.05)    low=low+1

With steps above, we can determine the significant spatial variation between areas and identify which district has significant higher or lower accessibility than others. According to the results of “High” and “Low”, accessibility is classified into different levels and spatial units within the same level are not significantly different. If the accessibility in one subdistrict is significantly higher than many other districts, we can infer that this subdistrict is at the level of good accessibility to hospitals compared with others. On the contrary, if accessibility in one subdistrict is significantly lower than many other subdistricts, it is at the level of poor accessibility. When subdistricts whose count of “High” and “Low” are both zero, that is, the accessibility within are neither significantly higher or lower to other subdistricts, we identify them at the level of mid-level accessibility. Those subdistricts that show both significantly higher and lower accessibility to others, that is, being significantly higher than some subdistricts (usually, subdistricts with extremely low accessibility) as well as being significantly lower than some other subdistricts (usually, subdistricts with extremely high accessibility), is also classified into the level of midlevel accessibility. The spatially adjusted ANOVA and multiple comparison procedure are compared with the traditional non-spatial one.

## 3. Results

### 3.1. Administrative District Scale

#### 3.1.1. Accessibility Measurements and Preprocessing

**Figure 6:**
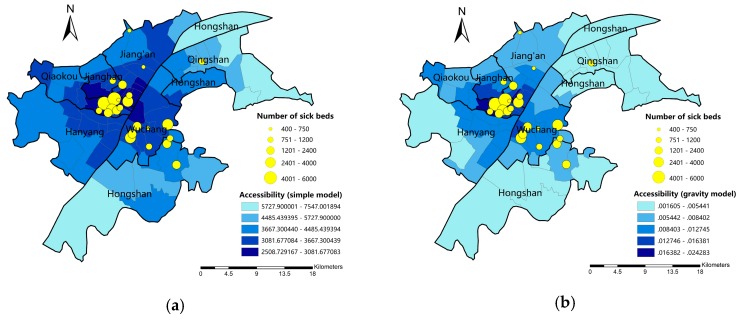
The accessibility calculated by two models on administrative district scale: (**a**) simple model; (**b**) gravity model.

On administrative district scale, the accessibility to hospitals is measured by two different models using travel time as impedance factor. Figure 6 shows the accessibility of 86 districts calculated by simple model and gravity model. Nature break method is used to classify the accessibility into 5 grades. The deeper the color, the better the accessibility to hospitals is. The results of simple model and gravity model differ from each other, especially in the peripheral study area. A majority of subdistricts show higher rank of accessibility in simple models. As a simple model can only reflect basic public transit service to hospitals, it could be assumed that the basic public transit service to hospitals is generally convenient. For gravity model, it takes demand scale and hospital serviceability into consideration. For some subdistricts that near to hospitals, the large population within might result in a short supply of hospital service.

Before using ANOVA for spatial difference analysis, normality test and data transformation should be performed. Table 1 demonstrates the results of Shapiro-Wilk normality test before and after data transformation. It is shown that the accessibility data are not normally distributed, “Box-Cox” power transformation is applied for data transformation. After power transformation with parameter $\alpha_{1}=-1.07$ (simple model) and $\alpha_{1}=0.222$ (gravity model), the accessibility data are turned into normally distributed one (*p*-value > 0.05). The result of Levene’s test showed that accessibility data are homoscedastic (*p*-value > 0.05). Therefore, ANOVA could be applied for spatial difference analysis.

#### 3.1.2. Spatial Autocorrelation Analysis and Elimination

As spatial dependency could affect the result of ANOVA, it is important to examine if spatial autocorrelation exists in experimental data. If accessibility data calculated by simple and gravity model is spatially autocorrelated, steps in Section 2.3.3 are conducted to filter out the influence of spatial autocorrelation. The Moran’s I regression model residuals are shown in Table 2. As the residuals in traditional liner regression model is significantly spatial autocorrelated (*p*-value < 0.05), spatial lag model is applied. The spatial autocorrelation parameter ρ for simple model and gravity model estimated in spatial lag model is 0.826 and 0.839 respectively. With *p*-value > 0.05 in spatial lag model, the spatial autocorrelation in residuals is eliminated and therefore could yield out spatially adjusted ANOVA.

#### 3.1.3. Spatial Difference Analysis and Multiple Comparison

The results for spatially adjusted ANOVA and traditional ANOVA are presented in Table 3. It can be seen that the F-value in traditional model is much larger than the spatial adjusted model, with *p*-value in the traditional model much smaller than it is in the spatially adjusted one. As *p*-values are both much smaller than 0.001, the accessibility data shows significant difference between seven administrative districts. Scheffe’s method is applied to further identify the differences between groups and the results are presented in Table 4. Before filtering out the influence of spatial autocorrelation, there are much more comparison pairs showed significant difference (10 pairs in simple model and 11 pairs in gravity model) than it is after the spatial adjusted procedure (4 pairs in simple model and 3 pairs in gravity model). 

**Figure 7:**
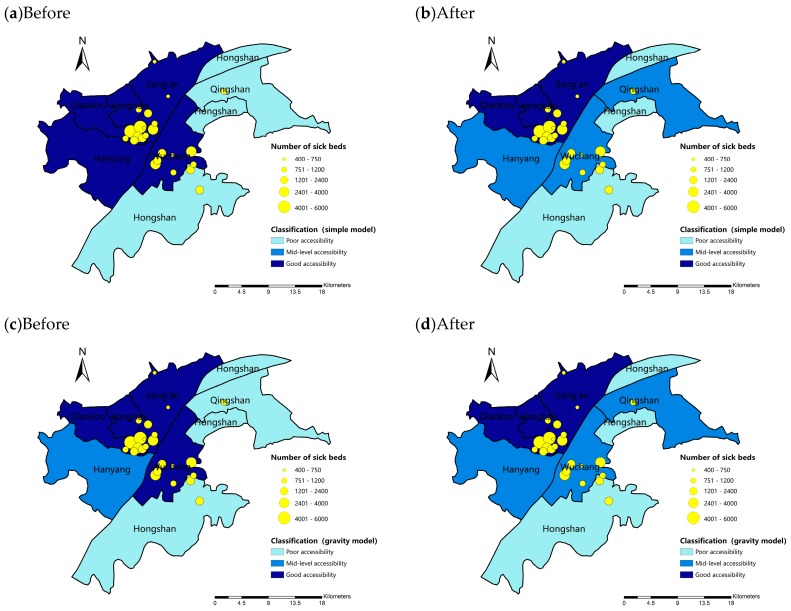
Classifications of accessibility to hospitals on administrative scale: (**a**,**b**) simple model; and (**c**,**d**) gravity model.

The corresponding accessibility level is presented in Figure 7. Districts within the same level show no significant difference between each other. In simple model, before filtering out the influence of spatial autocorrelation, accessibility to hospitals in Hongshan and Qingshan district are significantly lower than other 5 districts (Figure 7a). However, after the spatially adjusted procedure, the accessibility to hospitals in Hongshan district is only significantly lower than 3 districts (Jiang’an, Jianghan, Qiaokou). Accessibility in Wuchang Hanyang and Qingshan are at the level of mid-level accessibility, that is, the accessibility to hospitals within are neither significantly higher nor lower than other districts (Figure 7b). In gravity model, before filtering out spatial autocorrelation, Hongshan and Qingshan district are at the level of poor accessibility, Hanyang is at the level of mid-level accessibility and the rest of 4 districts are at the level of good accessibility (Figure 7c). While after filtering out the influence of spatial autocorrelation (Figure 7d), the results are same with those in simple model (Figure 7b).

It can be seen that before filtering out spatial autoclrrelation, more districts tend to show significantly lower accessibility than others and are classified at the level of poor accessibility. While after filtering out the influence of spatial autocorrelation, only Hongshan district is at the level of poor accessibility, which means the overall hospital accessibility in Hongshan districts is poor though there may be some subdistricts within have relativly high accessibility to hospitals. However, as there are only 7 study groups, the results are relatively obscure and can only reflect the spatial difference on a macro scale. Therefore, experiments are conducted on subdistrict scale to discover more detailed results.

### 3.2. Subdistrict Scale

#### 3.2.1. Accessibility Measurements and Preprocessing

**Figure 8:**
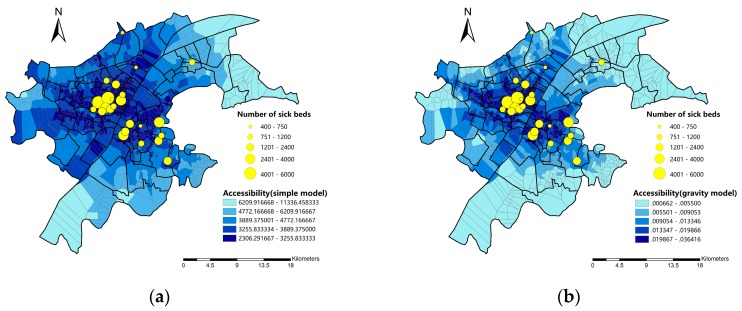
The accessibility calculated by two models on subdistrict scale: (**a**) simple model; (**b**) gravity model.

The results for accessibility measured by simple model and gravity model are presented in Figure 8. Similar to the results in administrative district scale, in simple model a large number of spatial units have a relatively higher accessibility (Figure 8a). Some spatial units that are not near to hospitals also show high accessibility to hospitals. We assume that the convenient route design of corresponding bus stops to hospitals might compensate the impedance of distance. In gravity model, however, less spatial units show the highest level of accessibility to hospitals (Figure 8b). That is, when taking demand and supply scale into consideration, the medical resources might not be equally distributed.

Shapiro-Wilk test and Levene’s test should be performed to identify if accessibility data is normally distributed and homoscedastic. As the accessibility data is not normally distributed (*p*-value < 0.05) and cannot be transformed into normal one, Kruskal-Wallis test is applied for the following spatial difference analysis.

#### 3.2.2. Spatial Autocorrelation Analysis and Elimination

The Moran’s I of regression model residuals are shown in Table 5. With *p*-value < 0.05, the accessibility calculated by simple model and gravity model is spatially autocorrelated. Using spatial lag model to estimate spatial autocorrelation parameter and the result show that $\rho_{1}$ = 0.8879 (simple model) and $\rho_{2}$ = 0.852 (gravity model). Follow the steps in Section 2.3.3 to eliminate the influence of spatial autocorrelation. With *p*-value > 0.05 in spatial lag model, the spatial autocorrelation in residuals is eliminated, yielding spatially adjusted Kruskal-Wallis test.

#### 3.2.3. Spatial Difference Analysis and Multiple Comparison

The *p*-value in Kruskal-Wallis test is smaller than 0.05 in both two models, which means the difference of accessibility between 86 subdistricts is significant. Dunn’s test is applied for multiple comparison to identify which groups differ. The output results of Dunn’s test in software R is an 86*86 symmetric matrix D, denoting the *p*-value of pairwise multiple comparison.

**Figure 9:**
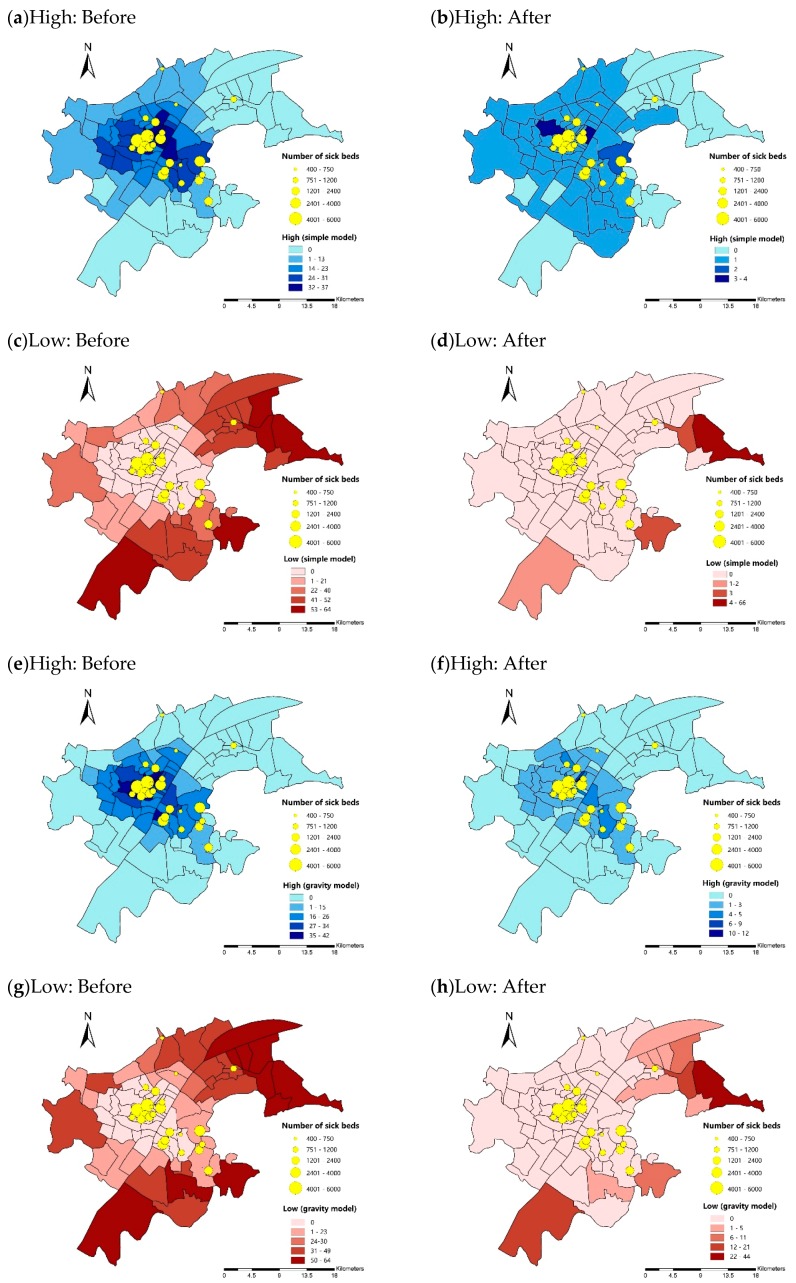
Number of “High” and “Low” before and after filtering out the influence of spatial autocorrelation: (**a**–**d**) simple model; and (**e**–**h**) gravity model.

After multiple comparison procedure described in Algorithm 1, the number of “High” and “Low” are presented in Figure 9. Similar to the results under administrative district scale, more comparison pairs showed significant difference in accessibility when the influence of spatial autocorrelation is not filtered out. In simple model, before filtering out the influence of spatial autocorrelation (Figure 9a,c), 17.4% of subdistricts show significant higher accessibility than other 37% of subdistricts and 22% of subdistrict showed significant lower accessibility than other 46.5% of subdistricts. After filtering out the influence of spatial autocorrelation (Figure 9b,d), only 4.7% of subdistricts show significantly higher accessibility than more than 1 district. 72.1% of subdistricts only showed significant higher accessibility than Baiyushan subdistrict. In gravity model, before filtering out spatial autocorrelation, the multiple comparison results presented in Figure 9e,g are similar to Figure 9a,c. However, after filtering out spatial autocorrelation (Figure 9f,h), less subdistricts showed significantly higher accessibility and more subdistricts show significant lower accessibility when comparing with the results in simple model(Figure 9b,d).

**Figure 10:**
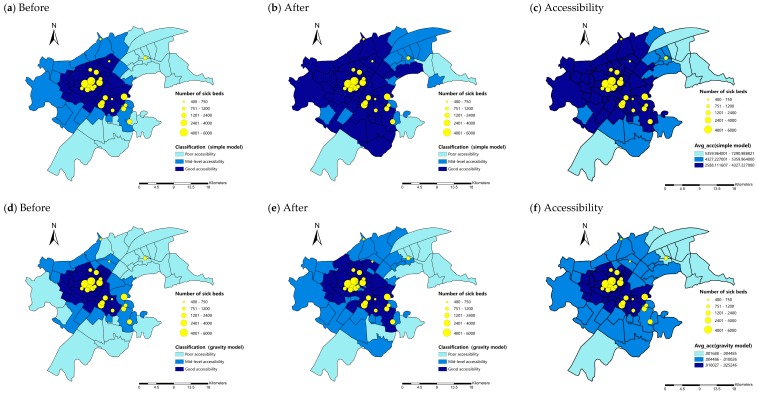
Classifications of accessibility to hospitals on subdistrict scale before and after filtering out the influence of spatial autocorrelation: (**a**,**b**) simple model; and (**d**,**e**) gravity model and corresponding average accessibility maps(**c**,**f**).

The final classification of accessibility level among subdistricts are presented in Figure 10. In simple model, before filtering out spatial autocorrelation, there are 23.3% subdistricts are at the level of poor accessibility, 19.8% at mid-level accessibility and 56.9% at the level of good accessibility (Figure 10a). However, after filtering out the influence of spatial autocorrelation, only 4 (4.7%) subdistricts including Qingling, Guanshan, Changqian, and Baiyushan are at the level of poor accessibility. The percentage of subdistricts with good accessibility reached to 76.7% and the remaining 18.6% is at mid-level of accessibility (Figure 10b). While in gravity model, before filtering out spatial autocorrelation, 27.9% of subdistricts are at the level of poor accessibility, 20.9% at the level of mid-level accessibility and 51.2% at the level of good accessibility (Figure 10d). After filtering out the influence of spatial autocorrelation, the percentage of subdistricts at the level of poor accessibility decrease to 14%. Meanwhile, 34.8% of subdistricts are at mid-level accessibility and 51.2% subdistricts are at the level of good accessibility. 

For better visualization, the accessibility calculation results (Figure 8a,b) are averaged by subdistrict units and the maps are shown in Figure 10c,f. It can be seen that the classification results after filtering out spatial autocorrelation (Figure 10b,e) are more in common with these accessibility calculation results. Besides, some previous studies also concluded that subdistrict with relatively lower accessibility to hospitals mainly distributed in west of Qingshan and south of Hongshan [38,39], which also indicate that the traditional non-spatial model’s result is inappropriate. When the spatial autocorrelation exists, owing to the variance inflation, more comparison pairs showed significant difference when none indeed exists. Therefore, in both simple and gravity model, some subdistricts showed significantly lower accessibility than others when they are indeed not and tends to be misclassified into the level of poor accessibility.

## 4. Discussion

### 4.1. Spatial Difference Analysis Based on ANOVA

In this paper, we developed a spatially adjusted ANOVA method in hospital accessibility analysis by using spatial lag regression model to estimate spatial autocorrelation parameter and filter out the spatial autocorrelation in residuals. If overall accessibility showed significant difference, multiple comparison methods are used to identify which study group indeed have significantly higher or lower accessibility than others. This spatially adjusted procedure could reduce the influence of variance inflation in ANOVA and, in return, decreases the chance of false rejection to null hypothesis [24,25,40]. 

By comparing the multiple comparison results before and after considering the influence of spatial autocorrelation, it can be concluded that the *p*-value in traditional ANOVA and its following multiple comparison procedures are much smaller, making more comparison pairs show significant difference. The classification results also support that the spatial adjusting procedure performs better than the traditional ones by being closer to the accessibility calculation results.

The accessibility to hospitals is analyzed on two scales to discover the spatial variations by regions in macro and micro dimensions. On administrative scale, there are 86 minimum spatial units (subdistricts) within 7 study groups (administrative districts). According to the results of spatial adjusting procedures, we found that accessibility to hospitals in Hongshan district is the worst. Although there are many bus stops and two 3A hospitals distributing in Hongshan, as the largest administrative district in Wuhan, Hongshan covers an area of 570 km^2^ and lies beside East Lake, which makes it less convenient for residents to get to 3A hospitals by public transport. 

For further detection of the spatial variations on a micro scale, 86 subdistricts are taken as study groups. The results for spatial difference analysis at subdistricts level are consistent to those at administrative districts level and reveal more details. Subdistricts with good accessibility to hospitals mostly aggregate in Wuchang, Qiaokou, Jianghan and Jiang’an district. Especially, Qiaokou and Jianghan district have a majority of subdistricts with good accessibility to hospitals. Subdistricts whose accessibility are significantly lower to others mainly distribute in Hongshan and Qingshan district (Qingling, Guanshan, Changqian, and Baiyushan in simple model; Tianxingxiang, Shizishan, Heping, Qingling, Guanshan, Xingouqiao, Honggangcheng, Gongrencun, Qingshanzhen, Changqian, Wudong, and Baiyushan in gravity model). These subdistricts are either severely lack of public transit resources to hospitals or have a very high number of populations. To improve the accessibility within these subdistricts, the government should make some policies to allocate health care resources. For example, setting funds to support to expand the number of sickbeds in 3A hospitals nearby such as WISCO general Hospital, Hubei Maternal and Child Health Hospital and Hubei Cancer Hospital, and therefore enlarging hospitals’ service capacity and scope. Besides, public transit department could add some convenient transit routes to hospitals in these subdistricts to decrease the travel time impedance. Moreover, telemedicine systems could be developed to facilitate high-quality medical services in these places [41]. In later urban design, policy makers should also pay attention to prevent the over-clustering of 3A hospitals [42]. To first solve the problem in poor accessibility areas could better achieve the goal of “leaving no one behind” and alleviate the existing inequality.

### 4.2. Comparison Between Simple Model and Gravity Model

Simple model and gravity model are used for accessibility measurements. According to the accessibility measurement results, we can see that a majority of spatial units show good accessibility in simple model, which means the basic public transit service to hospitals is generally good. However, as gravity model takes the opportunity of getting medical care into consideration, less spatial units show the highest level of accessibility to hospitals. 

On administrative district scale, the results between simple model and gravity model are generally the same. This is reasonable because there are only seven study groups so that we cannot determine which model can better reflect the spatial variations. However, on subdistrict scale, results are largely different. In simple model only 4.7% of subdistricts show poor accessibility and 76.7% of subdistricts show good accessibility. While in gravity model there are 51.2% of subdistricts at the level of good accessibility, 34.9% at the level of mid-level accessibility and 14% at the level poor accessibility. The results in simple model show that the overall basic public transit service to hospitals to central Wuhan is relatively equal and convenient. Although this result is consistent to accessibility measurement result, it is still questionable. As the biggest city in central China, Wuhan is developing rapidly in recent years and the inequality of accessibility to hospitals among districts is irresistible. 

Therefore, simple model may not be suitable for the spatial difference analysis to hospitals. It can only reflect basic public transit service to hospitals and over estimates the influence of hospitals that are far away. Besides, it cannot reflect the spatial differences in the perspective of getting medical opportunities. Gravity model is more appropriate because it not only considers the relationship between supply and demand scale but also puts distance decay function into the model to add reasonable weights to corresponding hospitals, thus can detect more hidden information about the inequal distribution of medical resources.

## 5. Conclusions

There are two major contribution of this paper, which could be summarized as follows. On the one hand, we put forward a spatial difference analysis method for accessibility to hospitals by regions using spatially adjusted ANOVA. This method could examine if accessibility to hospitals within different groups is significantly different or the differences of mean value are just due to unreasonable sampling. Comparing with the traditional ANOVA, the spatially adjusted ANOVA performs better by filtering out the influence of spatial autocorrelation, which largely avoids false rejection to null hypothesis (Type I error) caused by spatial dependency among sampling data. As the results of ANOVA show significant difference among study groups, corresponding multiple comparison methods are chosen to further identify which groups are significantly different. According to the multiple comparison result, accessibility to hospitals within study groups is classified into different levels and groups within the same level are not significantly different. This method could be employed not only in hospital accessibility assessment, but also in many other aspects (education, work, entertainment, etc.) to detect if these resources are equally distributed and find out which places are in significantly bad situation. It can shed some new lights in providing suggestions for urban planning and transit constructing.

On the other hand, the spatial difference analysis for accessibility to hospitals is conducted on two scales. One is administrative districts scale and another is subdistrict scale. The analysis in macro and micro dimension not only find out the overall poor or good accessibility areas but also point out regions with significantly lower accessibility to hospitals that needs to be improved at the first place.

However, some limitations still need to be considered. Firstly, in the calculation of hospital accessibility, some important factors such as residents’ preference of hospitals, different travel modes as well as their income are not taken into consideration [43,44,45]. Secondly, variations of accessibility in spatiotemporal patterns are not studied. Results of accessibility could vary during the course of the day or the week, but we do not investigate the fluctuation [30,46]. At the next step, we will consider these problems and conduct more comprehensive analysis.

## Figures and Tables

**Table 1:** Scheffe normality test before and after data transformation on administrative district scale.

*p*-Value	Simple Model	Gravity Model
before transformation	<0.0001	0.05
after transformation	0.076	0.12

**Table 2:** Moran’s I of regression model residuals on administrative district scale.

Accessibility	Liner Regression Model		Spatial Lag Model	
	Moran’s I	*p*-Value	Moran’s I	*p*-Value
Simple model	0.3796	<0.001	−0.032	0.6302
Gravity model	0.5158	<0.001	−0.0154	0.523

**Table 3:** ANOVA table for accessibility on administrative district scale: (a) simple model; (b) gravity model.

**(a) Simple Model**	**Degree of Freedom**	**Sum of Square**	**Mean Square**	**F-Value**	***p*-Value**
*Traditional ANOVA*					
Y	6	0.00000007	0.00000001	22.651	0.00000000
Residuals	79	0.00000004	0.0000000005		
*Spatially adjusted ANOVA*					
Y	6	0.00000006	0.0000000009	4.9269	0.0002504
Residuals	79	0.00000001	0.0000000002		
**(b) Gravity Model**	**Degree of Freedom**	**Sum of Square**	**Mean Square**	**F-Value**	***p*-Value**
*Traditional ANOVA*					
Y	6	0.110570	0.0184283	21.557	0.00000000
Residuals	79	0.067533	0.0008549		
*Spatially adjusted ANOVA*					
Y	6	0.0078432	0.0013072	4.5522	0.000489
Residuals	79	0.0226855	0.0002872		

**Table 4:** Results of multiple comparison on administrative district scale

		Simple Model				Gravity Model			
Comparison Pairs		Traditional		Spatially Adjusted		Traditional		Spatially Adjusted	
		Difference	*p*-Value	Difference	*p*-Value	Difference	*p*-Value	Difference	*p*-Value
Hongshan	Jiang’an	−0.00007265	0.0000 ***	−0.00002344	0.0113 **	−0.079888	0.0000 ***	−0.025619	0.0434 *
	Jianghan	−0.00008579	0.0000 ***	−0.00002507	0.0084 ***	−0.109038	0.0000 ***	−0.030125	0.0130 **
	Hanyang	−0.00005974	0.0000 ***	−0.00001970	0.1075	−0.068268	0.0001 ***	−0.020041	0.2943
	Wuchang	−0.00005962	0.0000 ***	0.00001865	0.1246	−0.074259	0.0000 ***	−0.023797	0.1069
	Qingshan	−0.00001597	0.8490	0.00000807	0.9347	−0.020734	0.8506	−0.010835	0.9088
	Qiaokou	−0.00007137	0.0000 ***	−0.00002056	0.0829 *	−0.102001	0.0000 ***	−0.030520	0.0172 **
Jiang’an	Jianghan	−0.00001453	0.8063	−0.00000163	1.0000	−0.029151	0.3212	−0.004506	0.9978
	Hanyang	0.00001152	0.9347	0.00000374	0.9996	0.011620	0.9814	0.005577	0.9936
	Wuchang	0.00001165	0.9238	0.00000478	0.9895	0.005628	0.9996	0.001822	1.0000
	Qingshan	0.0000553	0.0000 ***	0.00001537	0.2426	0.059154	0.0010 ***	0.014783	0.5672
	Qiaokou	−0.00001058	1.0000	0.00000288	0.9995	−0.022114	0.7125	0.004901	0.9973
Jianghan	Hanyang	0.00002605	0.2254	0.00000537	0.9956	0.040771	0.0723 *	0.010083	0.9023
	Wuchang	0.00002680	0.1986	0.00000642	0.9634	0.034779	0.1783	0.006328	0.9891
	Qingshan	0.00006983	0.0000 ***	0.00001700	0.1836	0.088304	0.0000 ***	0.019289	0.2872
	Qiaokou	0.00001442	0.8706	0.00000451	0.9954	0.007037	0.9992	−0.00040	1.0000
Hanyang	Wuchang	0.00000013	1.0000	0.00000105	0.9999	−0.005992	0.9996	−0.003756	0.9995
	Qingshan	0.00004378	0.0044 ***	0.00001263	0.5682	0.047534	0.0348 **	0.009206	0.9468
	Qiaokou	−0.00001163	0.9553	−0.00000014	1.0000	−0.033734	0.2792	−0.010478	0.9037
Wuchang	Qingshan	0.00004365	0.0036 ***	0.00001057	0.7399	0.053525	0.0079 ***	0.012962	0.7551
	Qiaokou	−0.00001176	0.9484	−0.00000191	1.0000	−0.027742	0.5035	−0.006723	0.9880
Qingshan	Qiaokou	−0.00005540	0.0001 ***	−0.00001248	0.6072	−0.081268	0.0000 ***	−0.019685	0.3111

Notes: *** significance at 1% level; ** significance at 5% level; * significance at 10% level.

**Table 5:** Moran’s I of regression model residuals on subdistrict scale.

Accessibility	Liner Regression Model		Spatial Lag Model	
	Moran’s I	*p*-Value	Moran’s I	*p*-Value
Simple model	0.49573	<0.0001	−0.07436	1
Gravity model	0.39730	<0.0001	−0.073657	1
